# Transcranial Direct Current Stimulation Modulates Cortical Neuronal Activity in Alzheimer's Disease

**DOI:** 10.3389/fnins.2016.00134

**Published:** 2016-03-31

**Authors:** Sara Marceglia, Simona Mrakic-Sposta, Manuela Rosa, Roberta Ferrucci, Francesca Mameli, Maurizio Vergari, Mattia Arlotti, Fabiana Ruggiero, Elio Scarpini, Daniela Galimberti, Sergio Barbieri, Alberto Priori

**Affiliations:** ^1^Fondazione IRCCS Ca' Granda Ospedale Maggiore Policlinico, Clinical Center for Neurostimulation, Neurotechnology, and Movement DisordersMilano, Italia; ^2^Dipartimento di Ingegneria e Architettura, Università degli Studi di TriesteTrieste, Italia; ^3^Istituto di Bioimmagini e di Fisiologia Molecolare, Consiglio Nazionale delle RicercheSegrate, Italia; ^4^Dipartimento di Ingegneria Elettrica e dell'Informazione “Guglielmo Marconi,” Università di BolognaCesena, Italia; ^5^Unità di Neurologia, Dipartimento di Fisiologia Medico-Chirurgica e dei Trapianti, Università degli Studi di Milano, Fondazione IRCCS Ca' Granda Ospedale Maggiore PoliclinicoMilano, Italia; ^6^Dipartimento di Scienze della Salute, Università degli Studi di Milano, Polo Ospedaliero San PaoloMilano, Italia

**Keywords:** transcranial Direct Current Stimulation (tDCS), neuromodulation, Alzheimer's disease, quantitative EEG, coherence, spectral analysis

## Abstract

Quantitative electroencephalography (qEEG) showed that Alzheimer's disease (AD) is characterized by increased theta power, decreased alpha and beta power, and decreased coherence in the alpha and theta band in posterior regions. These abnormalities are thought to be associated with functional disconnections among cortical areas, death of cortical neurons, axonal pathology, and cholinergic deficits. Since transcranial Direct Current Stimulation (tDCS) over the temporo-parietal area is thought to have beneficial effects in patients with AD, in this study we aimed to investigate whether tDCS benefits are related to tDCS-induced changes in cortical activity, as represented by qEEG. A weak anodal current (1.5 mA, 15 min) was delivered bilaterally over the temporal-parietal lobe to seven subjects with probable AD (Mini-Mental State Examination, MMSE score >20). EEG (21 electrodes, 10–20 international system) was recorded for 5 min with eyes closed before (baseline, t0) and 30 min after anodal and cathodal tDCS ended (t1). At the same time points, patients performed a Word Recognition Task (WRT) to assess working memory functions. The spectral power and the inter- and intra-hemispheric EEG coherence in different frequency bands (e.g., low frequencies, including delta and theta; high frequencies, including alpha and beta) were calculated for each subject at t0 and t1. tDCS-induced changes in EEG neurophysiological markers were correlated with the performance of patients at the WRT. At baseline, qEEG features in AD patients confirmed that the decreased high frequency power was correlated with lower MMSE. After anodal tDCS, we observed an increase in the high-frequency power in the temporo-parietal area and an increase in the temporo-parieto-occipital coherence that correlated with the improvement at the WRT. In addition, cathodal tDCS produced a non-specific effect of decreased theta power all over the scalp that was not correlated with the clinical observation at the WRT. Our findings disclosed that tDCS induces significant modulations in the cortical EEG activity in AD patients. The abnormal pattern of EEG activity observed in AD during memory processing is partially reversed by applying anodal tDCS, suggesting that anodal tDCS benefits in AD patients during working memory tasks are supported by the modulation of cortical activity.

## Introduction

Alzheimer's disease (AD) is a neurological disorder characterized by memory loss, severe intellectual impairment, and widespread cortical atrophy mainly localized in temporal-parietal (TP) lobe (Guze et al., 1991; Scarpini and Cogiamanian, 2003; Migliaccio et al., 2012). Morphological and functional data point out an early involvement of the temporal mesial areas followed by a progressive spread to the fronto-temporo-parietal areas with relative maintenance of the primary motor cortex (Kesslak et al., 1991; Braak et al., 1996; Karas et al., 2003). Functional neuroimaging studies showed a decreased metabolic activity in these areas (Haxby et al., 1987; Biegon et al., 1994; De Santi et al., 2001; Ewers et al., 2011). Brain tissue in AD patients is characterized by an increase of oxidative stress (OxS), with damage to proteins, lipids, and DNA oxidation/glycoxidation processes (Feng and Wang, 2012). OxS is generally an imbalance in production of Reactive Oxygen Species (ROS) and Reactive Nitrogen Species (RNS) vs. the antioxidant defense system. OxS caused by excessive production of ROS, primarily superoxide anion, is considered the most important mechanism by which risk factors deprive the endothelium of Nitric Oxide (NO) (Alusik et al., 2008). AD is hence characterized by a decreased concentration of NO (Selley, 2003; Guix et al., 2005) that is thought to contribute to cognitive decline (Katusic and Austin, 2014).

The application of transcranial Direct Current Stimulation (tDCS), a non-invasive technique for focal modulation of brain and nerve function (Nitsche and Paulus, 2000, 2001; Priori, 2003; Paulus, 2004; Wassermann and Grafman, 2005), over the TP brain areas provided encouraging results on memory improvement in patients with AD and was proposed as adjuvant therapy for AD (Ferrucci et al., 2008; Boggio et al., 2012). The facilitating effect of anodal tDCS is believed to improve the TP hypoactivation in AD patients, thus enhancing memory performances (Ferrucci et al., 2008). However, neither our previous work nor the other literature on tDCS in AD assessed tDCS effects at the neurophysiological level.

Quantitative electroencephalography (qEEG) consists in the application of mathematical algorithms to the EEG signal, aimed at highlighting “quantitative” information not available in “qualitative” (or paper-based) EEG analysis. In particular, EEG analysis in the frequency domain (power spectral analysis) provides information about the presence of different oscillations in the EEG that reflect the general arousal levels in the brain. Coherence analysis can be used to evaluate cortical connections and to provide additional sources of information about the topography of synchronous oscillatory activity (Locatelli et al., 1998; Anghinah et al., 2000; Stevens et al., 2001; Fonseca et al., 2015). qEEG is now well established for assessing the functional state of the brain (Gudmundsson et al., 2007), and for supporting the discrimination of different pathologies (Koberda et al., 2013).

In children with autism, the application of tDCS induced an improvement in the health/behavior and social domains as measured by the autism treatment evaluation checklist (ATEC), that was reflected in an improvement of the cortical activity pattern measured by EEG (Amatachaya et al., 2015). This study suggests that EEG analysis can provide a significant contribution for understanding tDCS-induced neurophysiological changes correlated to tDCS-induced clinical changes.

The neurophysiological cortical pattern of AD was studied since 1980s (Klimesch, 1999). Whereas, theta (2.5–7 Hz) oscillations (i.e., low-frequency activity) appear to be higher in AD patients than in normal subjects in TP areas, alpha (8–12 Hz) and beta (13–25 Hz) oscillations (i.e., high-frequency activity) are lower in AD patients in frontal and TP brain areas (Duffy et al., 1984; Chiaramonti et al., 1997; Jelic et al., 2000; Kramer et al., 2007; Koberda et al., 2013; Fonseca et al., 2015). Even though the definition of frequency band limits may vary according to the subject population (Klimesch, 1999), the alpha band power was positively associated with the search and retrieval mechanisms in long term memory whereas the theta band power was negatively associated with the information encoding in the hippocampo-cortical loops (Klimesch, 1999). In addition, a decreased alpha coherence was found with bipolar recordings in AD (Leuchter et al., 1992; Wang et al., 2014) particularly in the inter-hemispheric alpha coherence between occipital sites (Anghinah et al., 2000) and in temporo-parieto-occipital areas (Locatelli et al., 1998; Stevens et al., 2001; Jeong, 2004; Wang et al., 2014) thus suggesting that the alpha coherence decrease could be related to the lack of influence of subcortical cholinergic structures on cortical electrical activity. Also, Locatelli et al. (1998) reported an increase in delta coherence between frontal and posterior regions in AD patients, but only in a few patients, whereas others reported decreased theta coherence in the fronto and parieto-occipital areas (Wang et al., 2014). The decreased inter-hemispheric theta coherence correlates with lower Quality of Life indicators in AD patients than in controls (Fonseca et al., 2015).

Interestingly, the higher density of sources of theta, alpha, and beta activity were localized in the TP areas in AD patients whereas the source of these activities were more distributed in healthy controls (Vecchio et al., 2013). This suggests that applying tDCS over TP areas may have an effect also on the EEG pattern. Also, direct electric fields applied in endothelial cells culture were shown to increase NO production (Trivedi et al., 2013) thus suggesting that tDCS may change NO levels. In fact, models of the electric properties of the brain suggest that the electric field generated during tDCS in humans is around 1 mV/mm (Neuling et al., 2012) indicating that endothelial cell-dependent responses may be triggered during tDCS.

Because AD is characterized by impaired EEG pattern and decreased NO levels (Guix et al., 2005; Zhu et al., 2007; Katusic and Austin, 2014) and since tDCS is thought to affect both EEG and NO, in this work we investigated whether the effects of tDCS on memory functions in AD patients were consistent with tDCS-induced changes in EEG and NO levels, by analyzing a population of AD patients in which, in a previous work, we showed that anodal tDCS improved memory functions (Ferrucci et al., 2008).

## Methods

### Patients

We studied a subset of seven subjects (5 women and 2 men; mean ± SD age 75.4 ± 7.2 years; years of education 11.4 ± 5.5), from the patient set already considered in Ferrucci et al. (2008). The diagnosis of probable AD was based on the criteria of NINCDS-ADRDA (McKhann et al., 1984) and the DSM-IV. All patients were under treatment with cholinesterase inhibitors (ChEI). The Mini-Mental State Examination (MMSE) score was above 20 (22.4 ± 1.39). The study was carried out in accordance with the recommendations of the Ethical Committee of the Fondazione IRCCS Ca'Granda Ospedale Maggiore Policlinico with written informed consent from all subjects. All subjects gave written informed consent in accordance with the Declaration of Helsinki.

### EEG recordings and analysis

**Figure 1:**
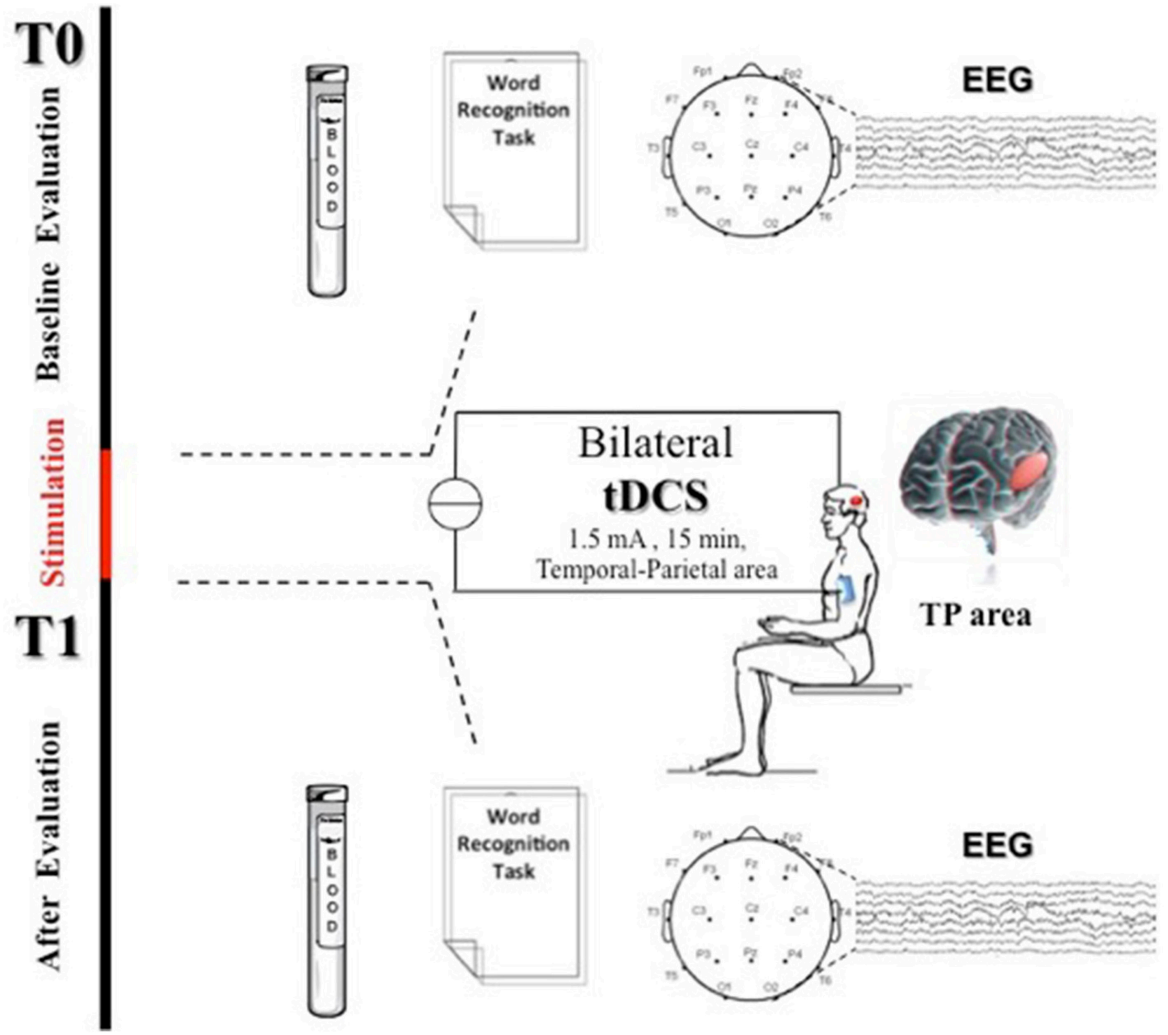
**Experimental protocol**. Transcranial Direct Current Stimulation (tDCS) was applied bilaterally for 15 min over the scalp in the TP areas (above P3-T5 left side and P6-T4 right side in according to the international 10–20 electrode placement system) at 1.5 mA. At baseline (t0) and 30 min after tDCS end (t1) patients were assessed through a word recognition task (WRT), EEG recording, and blood sample collection for NOx analysis.

EEG was recorded in a quiet room, with the subject awake, seated on a comfortable high-backed chair and with closed eyes, under healthcare personnel continuous control. Twenty-one electrodes (Ag/AgCl) were positioned according to the 10–20 International System using the EBNeuro Mizar-Light system (EBNeuro, Florence, IT). The sampling frequency was 1024 Hz with a bandpass of 0.5–500 Hz and a sensibility of 7 μV/mm. EEG was recorded for 5 min with eyes closed at baseline (t0) and 30 min (t1) after anodal tDCS (AtDCS) and cathodal tDCS (CtDCS) (Figure 1).

The software toolbox EEGLAB (Delorme and Makeig, 2004), running under the cross-platform MATLAB environment (The Math-Works 7.0, Inc.) was used for data processing. Preprocessing procedures included artifact rejection and filtering. EEG was analyzed in the frequency domain through power spectrum estimation. Power spectra were calculated with the Welch's averaged modified periodogram method (Welch, 1967) with a resolution of 1 Hz. Spectral power in the bands that were identified as neurophysiological biomarkers of AD were calculated for each subject before and after A- and CtDCS, namely delta (1–3 Hz), theta (4–7 Hz), alpha (8–12 Hz), and beta (13–35 Hz). The band definition (in terms of frequency interval) followed the classical EEG analysis. Spectral power was calculated on each electrode.

As a measure of synchronization between brain areas, coherence was estimated as:

$$\begin{array}{l} C_{xy}\left(f\right)=\frac{|P_{xy}\left(f\right){|}^{2}}{P_{xx}\left(f\right)P_{yy}\left(f\right)} \end{array}$$

Where *x* and *y* are two EEG signals from two different electrodes, P_*xx*_*(f)* and P_*yy*_(*f*) are the power spectral densities of *x* and *y*, and P_*xy*_*(f)* is their cross-spectral density.

In particular, inter-hemispheric EEG coherence in the frontal and antero-temporal (F-A-T) regions (Fp1-F7; Fp2-F8; Fp1-F3; Fp2-F4; Fp1-C3; Fp2-C4; F7-C3; F8-C4; F3-C3; F4-C4) and in the temporo-parieto-occipital (TP-O) regions (O1-P3; O2-P4; O1-T5; O2-T6; O1-C3; O2-C4; P3-C3; P4-C4; T5-C3; T6-C4) were calculated in the same frequency bands.

### tDCS and memory task

The full stimulation protocol is described in Ferrucci et al. (2008). tDCS was delivered at 1.5 mA intensity for 15 min over bilateral TP areas (above P3-T5 left side and P6-T4 right side according to the international 10–20 electrode placement system) through a commercial DC stimulator, connected to thick (0.3 mm) saline-soaked sponge electrodes, two placed over the scalp (active electrodes) and the other one (reference electrode) over the right deltoid muscle (for all the details, see Ferrucci et al., 2008). Each patient underwent two tDCS sessions, one for AtDCS and one for CtDCS stimulation, in a randomized order, with at least 1-week interval between the two sessions. tDCS polarity referred to the active electrodes over the scalp. The wide electrode surface (scalp electrode 25 cm^2^; deltoid electrode 64 cm^2^) avoided the possible harmful effects of high current density. To guarantee safety we applied to each stimulation site current at a density of 0.06 mA/cm^2^ and delivered a total charge of 0.054 C/cm^2^. These intensities are below the threshold for tissue damage (Liebetanz et al., 2009).

Before and after tDCS, recognition memory function was assessed by a pencil-and-paper word recognition task (WRT) over a set of 24 words (12 previously seen by the patients, and 12 randomly chosen from a word set), as fully described in our previous paper (Ferrucci et al., 2008). The difference between the words correctly recognized as previously seen (true positive) and those incorrectly recognized as previously seen (false positive) was considered for the analysis.

### Blood sample collection

A blood sample was collected to determine plasma levels of nitrite and nitrate (NO^2^ + NO^3^) both at baseline (t0) and 30 min (t1) after anodal and cathodal tDCS (Figure 1). Venous blood was drawn from the antecubital vein into a 10-mL EDTA vacutainer tube (Vacutainer, Becton Dickinson, USA). Plasma was immediately separated by centrifuge (5702R, Eppendorf, Germany) at 1000 × g for 10 min at 4°C. Total NO level (NOx) determination was performed using the Griess method with a commercial assay kit: Nitric Oxide (NO^2−^/NO^3−^) detection kit (Fisher Scientific, USA).

Samples were read by the addition of Griess reagents at 545 nm by a microplate reader spectrophotometer (Infinite M200, Tecan, Austria). The results were expressed in umol/L. All samples were determined in duplicate and the inter-assay coefficient of variation was in the range indicated by the manufacturer.

### Data analysis

As a first step, to establish the relationship between patient's cognitive condition and EEG measures, we verified whether baseline (t0) spectral powers in the area below the tDCS electrodes correlated with the patient's MMSE. We did the same with the NOx concentrations at baseline. Also, we verified the effect of AtDCS and CtDCS on the WRT task in our patients that were a subset of those described by Ferrucci et al. (2008).

We then assessed the effect of tDCS on both EEG spectral powers of the selected frequency bands (spectral power in a certain frequency band over a certain electrode) and coherences (coherence in a certain frequency band between two electrodes) by calculating the percentage changes of each variable between t0 (baseline) and t1 (30 min after tDCS) as:

(1) $$\begin{array}{r} \text{Delta}=\left(\text{v}\left(\text{t}1\right)-\text{v}\left(\text{t}0\right)\right)∕\text{v}\left(\text{t}0\right) \end{array}$$

Where v(t) is the value of the spectral or coherence variable at t0 and at t1.

For spectral powers, because the limits of the frequency bands can vary from patient to patient (Klimesch, 1999), while we calculated the spectral power in the classical EEG bands, we did not consider the bands as completely independent variables: we considered separately the “low-frequencies” (LF) (delta and theta) to cover the whole 2–7 Hz band, and the “high-frequencies” (HF) (alpha and beta), to cover the whole 8–25 Hz band. Also, we considered separately the electrodes over the frontal (Fp1, Fp2, F3, F4, F7, F8), TP (P3, P4, T3, T4, T5, T6), central (C3, C4, Cz), and occipital (O1, O2) areas.

Spectral powers and coherences were normally distributed (single-sample Kolmogorov–Smirnov tests *p* > 0.05), and therefore mean, standard error of the means, and parametric statistical analyses are presented. Hence, we used a four-way ANOVA with factors “electrode” (one level for each of the electrodes in the area), “side” (right and left), “band” (delta and theta for LF and alpha and beta for HF), and “stimulation” (AtDCS and CtDCS).

For coherences, we considered separately F-A-T and TP-O coherences and we run a two-way ANOVA with factors “electrode pair” (one level for each of the electrode pairs in the area) and “stimulation” (AtDCS and CtDCS).

Tukey's honest tests were used for *post-hoc* analysis. Probability levels of *p* < 0.05 were considered significant.

Finally, we assessed the correlation of tDCS-induced memory function improvement with the tDCS-induced EEG changes that resulted significant. To do so, Spearman's correlation coefficient (*p* < 0.05) was calculated between spectral powers or coherences that displayed significant tDCS-induced changes at t0 and t1 and the corresponding WRT result at t0 and t1 (after AtDCS or CtDCS).

Finally, to disclose the correlation between significant EEG changes and NOx changes, we calculated the Spearman's correlation coefficient (*p* < 0.05) between spectral power at t0 and t1 and the corresponding NO level at t0 and t1 (after AtDCS or CtDCS).

Throughout the text, values are expressed as mean ± standard errors of the mean (SE).

## Results

### Baseline evaluation and tDCS effect on memory functions

**Figure 2:**
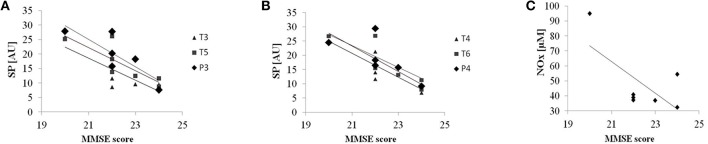
**Baseline correlations**. Correlation between Mini-Mental State Examination (MMSE score) and EEG spectral powers (SP) in the HF at baseline under the electrodes in left **(A)** and right **(B)** temporo-parietal areas. **(C)** Correlation between Mini-Mental State Examination (MMSE score) and NOx concentration (uM). Solid lines represent the linear regression fit.

Baseline patient's characteristics are reported in Ferrucci et al. (2008). We found an inverse correlation between MMSE scores and spectral powers in the HF under the electrodes in both the left (T3: *R*^2^ = 0.67, *p* = 0.024; T5: *R*^2^ = 0.67, *p* = 0.023; P3: *R*^2^ = 0.74, *p* = 0.012) (Figure 2A) and the right TP areas (T4: *R*^2^ = 0.75, *p* = 0.011; T6: *R*^2^ = 0.72, *p* = 0.015; P4: *R*^2^ = 0.62, *p* = 0.035) (Figure 2B). Even though not reaching significance (*R*^2^ = 0.46, *p* = 0.095), NOx baseline concentrations showed a similar trend: lower NOx levels are associated with higher MMSE scores (Figure 2C).

As previously reported on the full population (Ferrucci et al., 2008), AtDCS improved WRT results in all subjects (t0 vs. t1: 3.1 ± 0.8 vs. 5.6 ± 1.1, *p* = 0.015), whereas CtDCS tended to worsen it (t0 vs. t1: 4.5 ± 0.9 vs. 2.6 ± 1.1, *p* = 0.08). Table 1 reports individual WRT scores and NO concentrations. All the patients tolerated the procedure well, and did not experience adverse effects. None of them was able to distinguish AtDCS from CtDCS.

**Table 1:** **Individual results at the Word Recognition Task (WRT) and NO levels before (t0) and after (t1) AtDCS and CtDCS**.

**Subject**	**AtDCS**				**CtDCS**			
	**WRT**		**NO**_****x****_		**WRT**		**NO**_****x****_	
	**t0**	**t1**	**t0**	**t1**	**t0**	**t1**	**t0**	**t1**
1	2.0	2.0	95.0	105.3	3.0	2.0	77.2	74.9
2	1.0	4.0	36.8	36.2	0.0	0.0	38.1	35.5
3	4.0	10.0	39.5	39.5	6.0	5.0	61.0	65.6
4	1.0	3.0	38.9	39.5	7.0	6.0	28.7	31.9
5	2.0	4.0	54.4	53.8	7.0	0.0	47.7	44.0
6	5.0	8.0	38.8	28.4	5.0	5.0	39.1	34.4
7	7.0	8.0	32.4	34.1	4.0	0.0	50.6	52.7

### tDCS effects on EEG rhythms

**Figure 3:**
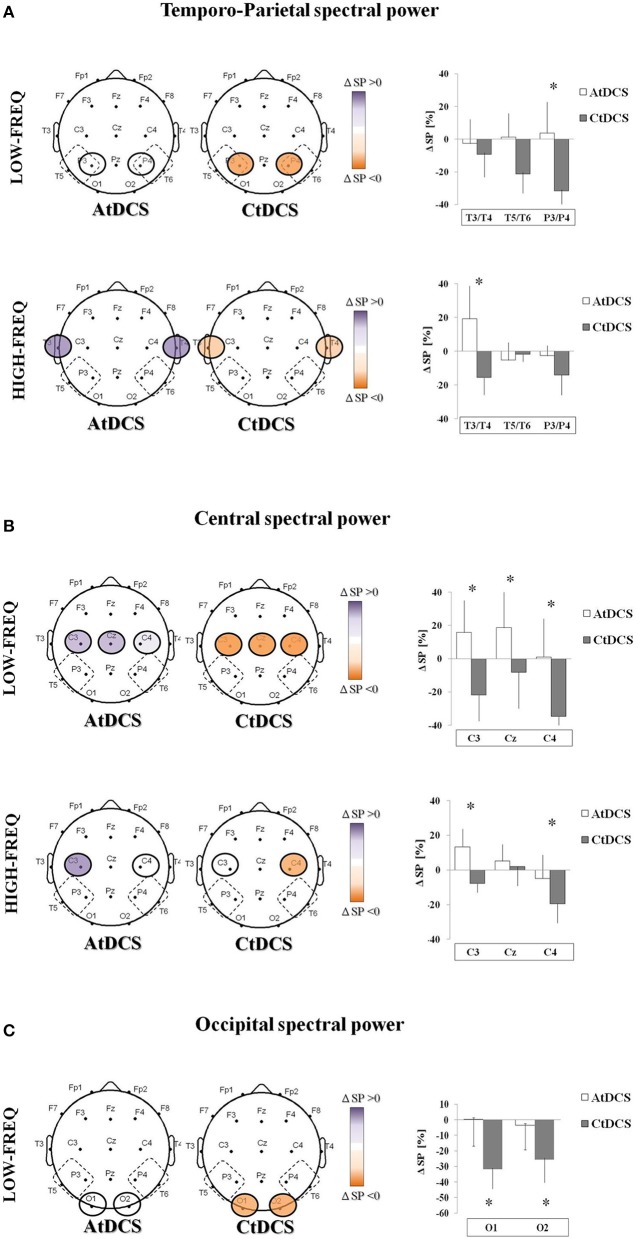
EEG spectral power (SP) changes induced by Anodal tDCS (AtDCS) and Cathodal tDCS (CtDCS) from statistical analysis (repeated measures analysis of variance ANOVA; ^*^p < 0.05). The graphical representation of the scalps displays the EEG channels with significant SP changes from *post-hoc* test. Violet represents SP increase and orange SP decrease from baseline. The histograms show the mean SP changes (*n* = 7) after AtDCS (white) and CtDCS (gray). On the y-axis is represented the SP percentage change from baseline and on the x-axis the EEG channels. Results are expressed as mean ± standard error (SE). **(A)** EEG SP changes induced by AtDCS and CtDCS in the Temporo-Parietal area in the low-frequency (delta and theta, upper panel) and high-frequency (alpha and beta, lower panel) bands: T3, T5, P3: left side; T4, T6, P4: right side. **(B)** EEG SP changes induced by AtDCS and CtDCS in the Central area in the low-frequency (delta and theta, upper panel) and high-frequency (alpha and beta, lower panel) bands: C3, Cz, C4. **(C)** EEG SP changes induced by AtDCS and CtDCS in the Occipital area in the low-frequency (delta and theta, upper panel) and high-frequency (alpha and beta, lower panel) bands: O1, O2.

In frontal areas, tDCS had no effects on EEG power neither in the LF nor in the HF. In TP areas, CtDCS significantly decreased LF below tDCS electrodes (P3 and P4, AtDCS vs. CtDCS: 3.7 ± 7.2% vs. −31.8 ± 4.3%, *p* = 0.03, Figure 3A upper panel), whereas AtDCS significantly increased HF (T3 and T4, AtDCS vs. CtDCS: 19.2 ± 7.4% vs. −5.2 ± 3.9%, *p* = 0.02, Figure 3A lower panel). In central areas, CtDCS significantly decreases LF below all electrodes (C3, AtDCS vs. CtDCS: 15.7 ± 7.3% vs. −21.9 ± 5.9%, *p* < 0.0001; C4, AtDCS vs. CtDCS: 1.04 ± 8.7% vs. −34.7 ± 3.8%, *p* < 0.0001; Cz, AtDCS vs. CtDCS: 18.6 ± 8.6% vs. −8.1 ± 8.3%, *p* < 0.0001; Figure 3B, upper panel). Conversely, AtDCS increased HF oscillations below C3, whereas CtDCS decreased them below C4 (C3, AtDCS vs. CtDCS: 13.3 ± 3.9% vs. −7.8 ± 1.9%, *p* = 0.0005; C4, AtDCS vs. CtDCS: −4.8 ± 5.2% vs. −19.4 ± 4.2%, *p* = 0.007; Figure 3B, lower panel). As well as in the other areas, CtDCS increased LF in the whole occipital area (O1, AtDCS vs. CtDCS: 0.22 ± 6.5% vs. −31.9 ± 4.7%, *p* < 0.0001; O2, AtDCS vs. CtDCS: −3.43 ± 6.0% vs. −25.5 ± 5.6%, *p* < 0.0001; Figure 3C), whereas no effect was observed on HF.

### tDCS effects on EEG coherences

**Figure 4:**
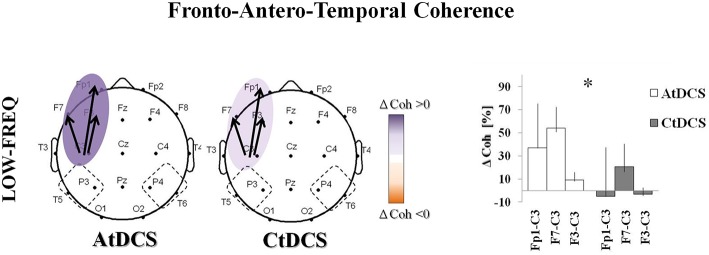
**EEG coherence (Coh) changes induced by Anodal tDCS (AtDCS) and Cathodal tDCS (CtDCS) in the Fronto-Antero-Temporalarea**. The panel shows the significant differences on EEG Coh (two-way repeated measure analysis of variance ANOVA; ^*^p < 0.05) for low-frequency (delta): Fp1-C3, F7-C3, F3-C3. The graphical representation of the scalps displays the significant difference on EEG Coh (Violet represents Coh increase and orange Coh decrease from baseline). The bold arrows show the significant differences at *post-hoc* test. The histograms represent the mean Coh changes (*n* = 7) after AtDCS (white) and CtDCS (gray). On the y-axis is represented the Coh percentage change from baseline and on the x-axis the EEG channels. Results are expressed as mean ± standard errors (SE).

**Figure 5:**
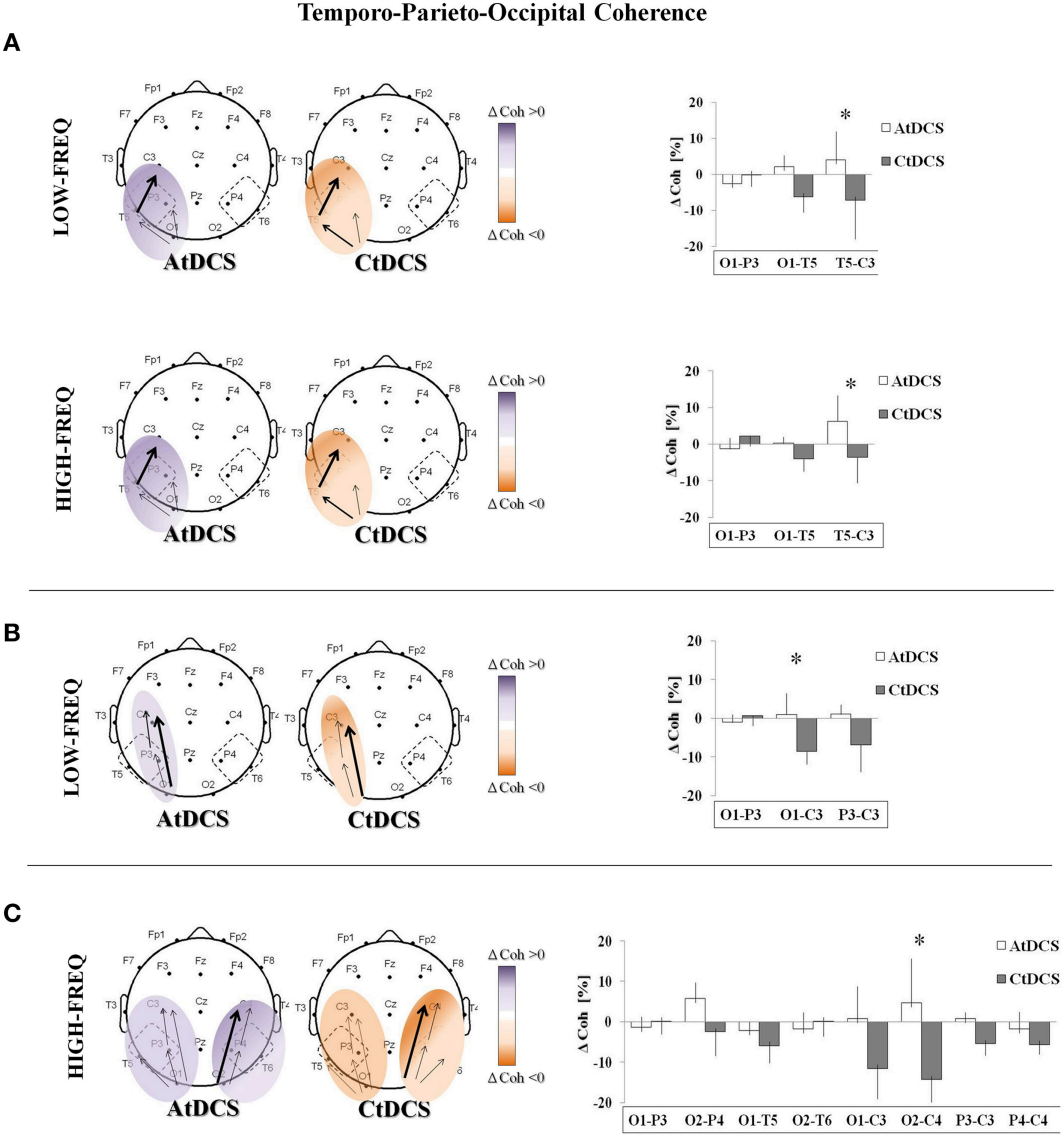
**EEG coherence (Coh) changes induced by Anodal tDCS (AtDCS) and Cathodal tDCS (CtDCS) in the Temporo-Parieto-Occipital area**. The panel shows the significant differences on EEG Coh (two-way repeated measure analysis of variance ANOVA; ^*^p < 0.05) for low-frequency (delta and theta, upper panel) and high-frequency (alpha and beta, lower panel): O1-P3, O1-T5, T5-C3 **(A)**; low-frequency (delta, theta): O1-P3, O1-C3, P3-C3 **(B)**; high-frequency (alpha, beta): O1-P3, O2-P4, O1-T5, O2-T6, O1-C3, O2-C4, P3-C3, P4-C4 **(C)**. The graphical representation of the scalps displays the significant difference on EEG Coh (Violet represents Coh increase and orange Coh decrease from baseline). The bold arrows show the significant differences at *post-hoc* test. The histograms represent the mean Coh changes (*n* = 7) after AtDCS (white) and CtDCS (gray). On the y-axis is represented the Coh percentage change from baseline and on the x-axis the EEG channels. Results are expressed as mean ± standard errors (SE).

We observed a significant effect of tDCS on the fronto-antero-temporal (Figure 4) and the temporo-parieto-occipital (Figure 5) coherences. AtDCS significantly increased the fronto-antero-temporal coherence in the LF oscillation (Fp1-C3, AtDCS vs. CtDCS: 36.8 ± 38.5% vs. −5.0 ± 42.6%; F7-C3, AtDCS vs. CtDCS: 54.2 ± 18.4% vs. 20.7 ± 19.6%; F3-C3 AtDCS vs. CtDCS 9.26 ± 6.6% vs. −3.2 ± 5.8%, *p* = 0.020%; Figure 4). Similarly, in the temporo-parieto-occipital area, AtDCS significantly increased both LF and HF coherences, whereas CtDCS decreased them. More specifically, AtDCS increased LF T5-C3 and O1-C3 cohenrences (T5-C3, AtDCS vs. CtDCS: 4.0 ± 7.9% vs. −7.1 ± 10.8%; *p* = 0.044; Figure 5A, upper panel; O1-C3, AtDCS vs. CtDCS: 0.9 ± 5.6% vs. −8.6 ± 4.6%; *p* = 0.034; Figure 5B) and it increased HF T5-C3 and O2-C4 coherences (T5-C3, AtDCS vs. CtDCS: 6.3 ± 7.1% vs. −3.6 ± 6.9%; *p* = 0.050; Figure 5A, lower panel; O2-C4, AtDCS vs. CtDCS: 4.7 ± 10.9% vs. −14.3 ± 6.9%; *p* = 0.009; Figure 5C).

### Correlation between tDCS effects on EEG and cognitive performance

**Figure 6:**
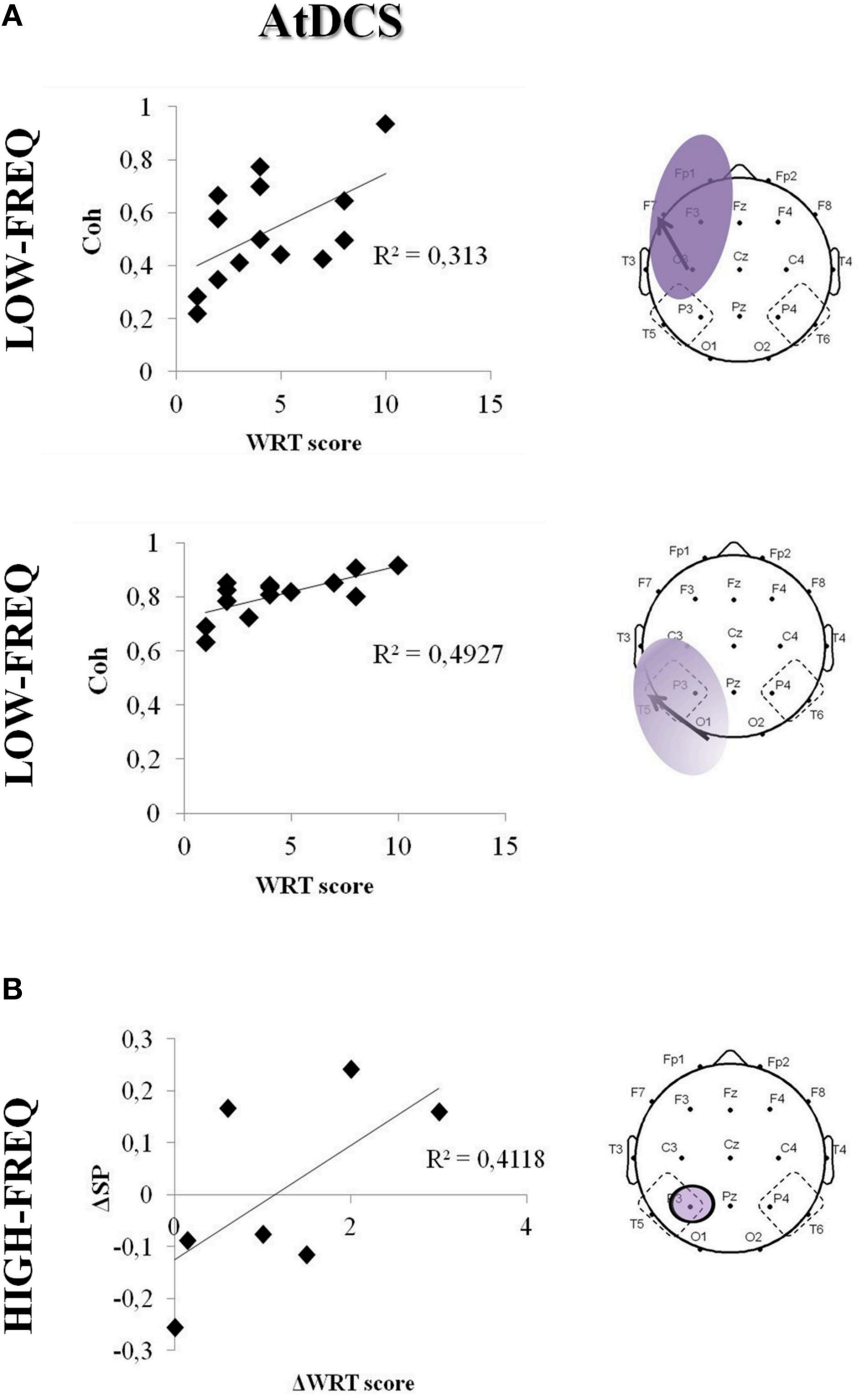
**Correlation between cognitive task performance (ΔWRT score) and EEG coherence (Coh) in the LF (A) and HF (B) in the fronto-central area/temporo-parieto-occipital after AtDCS**. The bold arrows represent the coherences under examination. Single data are represented as diamonds. Solid lines represent the linear regression fit. The correlation coefficient (*r*^2^) is reported in each panel.

The boosting effect of AtDCS on LF coherences significantly correlated with the cognitive performance at the WRT task (F7-C3: *R*^2^ = 0.31, *p* = 0.037; O1-T5: *R*^2^ = 0.49, *p* = 0.012) (Figure 6A). Although not significant, we also observed a trend toward correlation between the WRT task performance and the increase of HF power in the TP area (P3: *R*^2^ = 0.41, *p* = 0.10) (Figure 6B).

**Figure 7:**
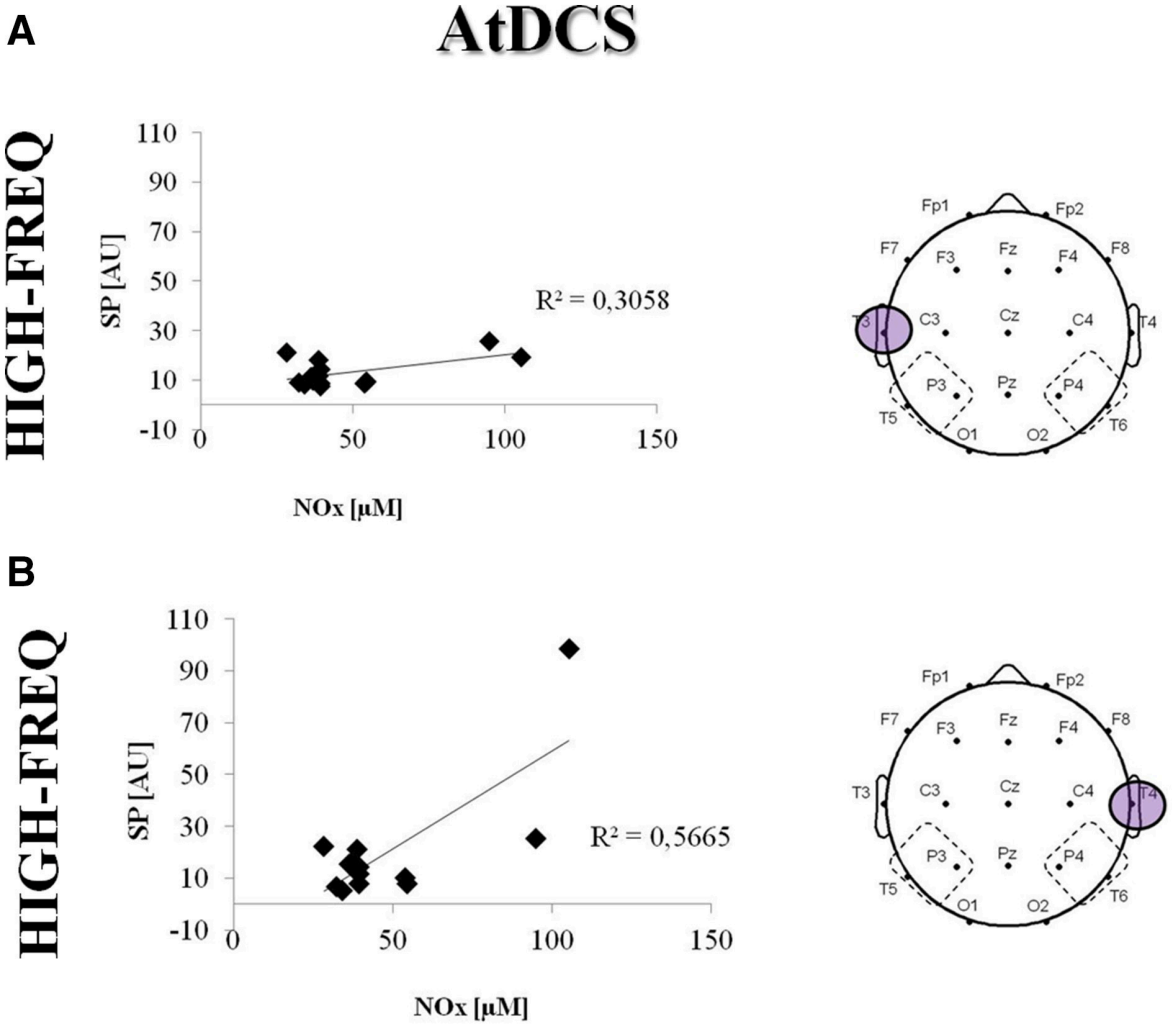
**Correlation between NOx concentration (uM) and Spectral Powers (SP, AU) in the HF under the electrodes T3 (A) and T4 (B) after AtDCS**. Single data are represented as diamonds. Solid lines represent the linear regression fit. The correlation coefficient (*R*^2^) is reported in each panel.

In addition, the HF power increase after AtDCS in the TP areas was directly correlated with an increase in the NOx levels observed in patients after AtDCS (T3: *R*^2^ = 0.30; *p* = 0.002; T4: *R*^2^ = 0.56; *p* = 0.012) (Figure 7).

## Discussion

**Figure 8:**
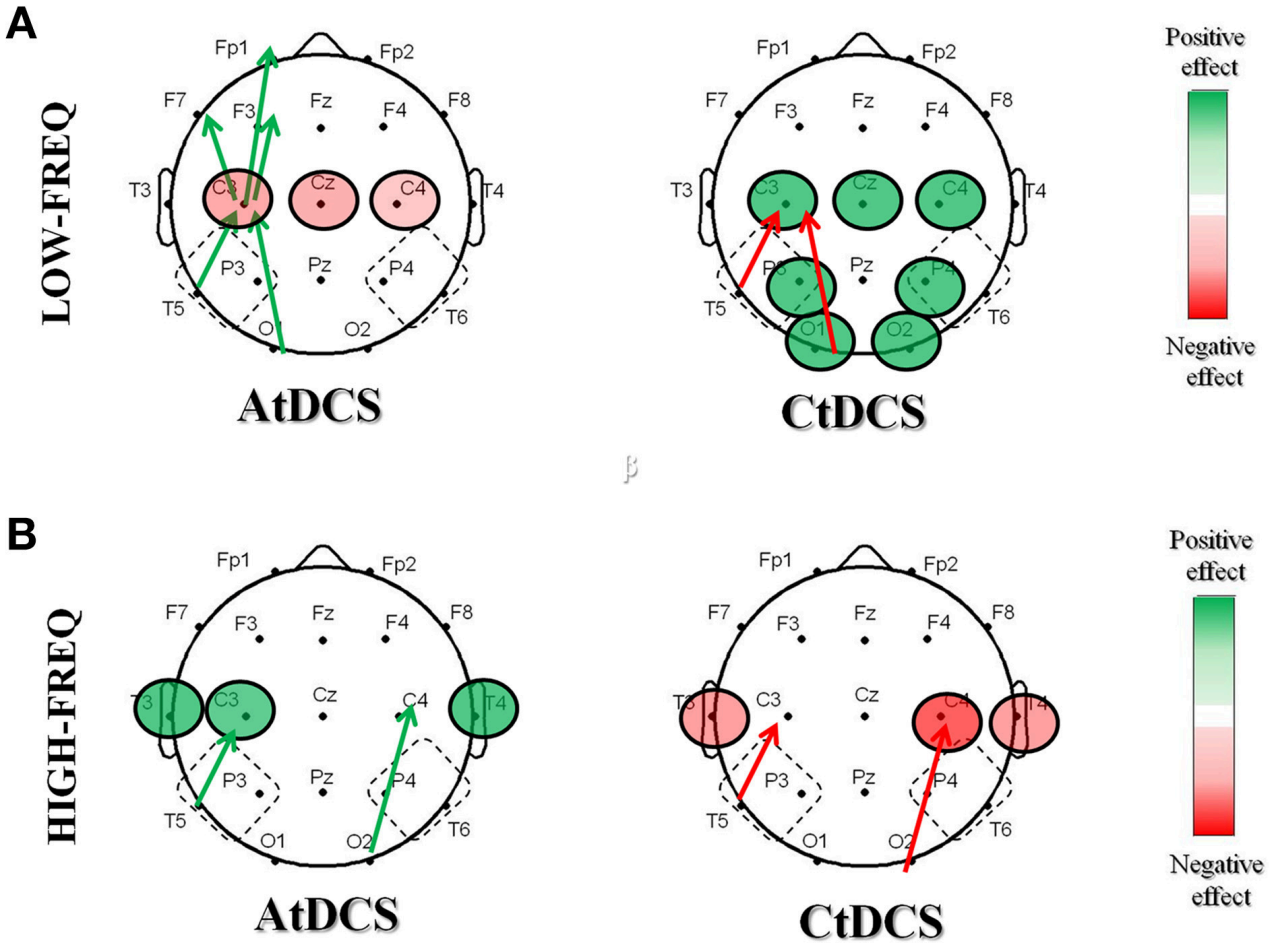
**Summary of the significant effects induced by AtDCS and CtDCS on the EEG pattern in the low frequency (A) and the high frequency (B) oscillations**. The graphical representation of the scalps displays the significant positive (green) and negative (red) effects on EEG spectral powers (circles) and coherences (arrows).

In this study, we investigated whether tDCS has an effect on EEG rhythms and coherences, and whether these changes could provide insights on the positive effects of tDCS on memory functions in AD. Our results showed that both A- and CtDCS are able to modulate cortical electrical activity as measured by qEEG and that the tDCS-induced modulations in EEG are consistent with the clinical effects of tDCS on memory in AD patients (Ferrucci et al., 2008; Boggio et al., 2009, 2012). More specifically, even though in a limited number of subjects, we observed that tDCS improves EEG patterns (Figure 8), both acting on the LF (delta and theta) and the HF (alpha and beta) oscillations. Whereas, CtDCS produces an unspecific positive decrease in the LF oscillations in the central-temporal-parietal-occipital areas, AtDCS has a more specific effect in the stimulation area, by increasing HF oscillations and coherences. Also, the effects of AtDCS on spectral powers and coherences correlate both with the improved clinical performance of the subjects at the WRT task and with the increased level of NOx following stimulation. These results suggest that the increased HF power and LF/HF coherences following AtDCS might be involved in the improved performance of AD patients at the memory task. The effect of CtDCS, despite being positive for the EEG pattern, has no correlation with the performance at the WRT task.

In fact, the EEG pattern of AD patients described in the literature suggests that decreased HF spectral powers in the frontal and TP areas can be involved in the long-term memory search and retrieval mechanisms (Klimesch, 1999; Koberda et al., 2013). This is consistent with our findings on the inverse correlation between patient's MMSE and basal HF spectral powers as well as on the direct correlation between HF increase and WRT improvement after AtDCS. Also, the literature shows that AD is characterized by abnormal decrease of inter- and intra- hemispheric EEG coherences that can be representative of AD widespread cerebral degeneration (Jiang, 2005), and may indicate an abnormal connectivity between cortical and subcortical structures (Locatelli et al., 1998; Vecchio et al., 2016). An increased demand of HF power and coherence in the temporal areas was observed in AD patients compared to controls during working memory workload (Hogan et al., 2003), possibly reflecting an enhanced efforts in patients than in controls. Hence, AtDCS, by increasing the HF power level and coherence in the TP areas, could respond to the increased demand in AD, thus improving WRT performance.

AtDCS, in our findings, also had an increasing effect on LF coherence, that was positively correlated with better performances at the WRT. These results complement previous observations that associated lower theta coherence with poorer quality of life indicators in AD patients than in controls (Fonseca et al., 2015).

On the other hand, our data showed that CtDCS has a widespread decreasing effect on the LF oscillation power not correlated to the WRT performance. However, the role of theta band in humans is still to be clarified: the increased theta power is not specifically associated with AD, but it was observed also in attention deficit disorders and in traumatic brain injuries (Koberda et al., 2013; Ulam et al., 2015). Even though the AD EEG pattern is characterized by an increased activity in the theta oscillation (Klimesch, 1999; Koberda et al., 2013), this pattern is not directly related to working memory processing (Hogan et al., 2003), thus possibly explaining why we did not find a correlation between the WRT performance and the decrease of theta power.

Our results are in agreement with previous findings on patients with traumatic brain injury, showing that tDCS-induced normalization of the EEG pattern correlates with better performances at neuropsychological tests (Ulam et al., 2015). In particular, authors report a decreased theta power and increased alpha power in frontal areas after AtDCS and suggest that the cumulative effect of consecutive tDCS sessions may regulate cortical excitability by normalizing frontal EEG pattern.

In addition to EEG features, we provided preliminary data on the correlation between neurobiological markers and the memory state: even though observed in the acute stage, higher NOx levels after tDCS correlated with both the positive effects of AtDCS on HF and on LF. Recent findings showed that tDCS has a role in the neurogenic control of the cerebral blood flow (Pulgar, 2015) that is directly related to the development of neurodegenerative diseases (Farkas and Luiten, 2001). Low electrical fields applied to endothelial cells produced increased NO levels (Trivedi et al., 2013), and, in turn, produce vasodilatation. These findings suggest that tDCS may act on NO to increase brain perfusion and improve memory performance.

Despite promising, our results suffer from the limited number of subjects treated with tDCS that claims for a study on a larger sample of AD patients. This implied that some trends in EEG and neurobiological markers did not reach statistical significance. Also, since the exact definition of EEG band limits in AD is variable across subjects (Klimesch, 1999), in our study, we decided to refer to LF oscillations, including delta and theta bands, and HF oscillations, including alpha and beta bands. Finally, in our subgroup of patients, to avoid subjecting participants to another long experimental session, we decided not to record sham EEG. This was in line with our aim, because we only wanted to investigate whether the effects of tDCS on memory were reflected by EEG pattern changes. Our results showed that A- and CtDCS have different effects on the electrical activity, thus ruling out the possibility that modifications in the EEG could be observed in any group after tDCS (i.e., the second time that EEG is measured). Our results are also supported by other findings proposing that each tDCS polarity can be considered as the best possible control for the other (Cogiamanian et al., 2008; Truini et al., 2011; Lamy and Boakye, 2013; Bocci et al., 2015).

In conclusion our results provided evidence that tDCS induces significant modulations in the cortical EEG activity in AD patients. The abnormal pattern of EEG activity observed in AD during memory processing is partially reversed by applying AtDCS, suggesting that AtDCS benefits in AD patients during working memory tasks are supported by the modulation of neuronal cortical activity.

## Author contributions

SM, SM-S, and MR designed and conducted the experiment, analyzed the data, interpreted the results, and drafted the manuscript. AP: designed the experiment, interpreted the results, and reviewed the manuscript. MA analyzed the data and reviewed the manuscript. RF, FM, FR conducted the experiment, interpreted the results, and reviewed the manuscript. MV, DG conducted the experiment, and reviewed the manuscript. ES, SB interpreted the results, and reviewed the manuscript. All the authors approved the final version of the manuscript.

## Funding

The study was supported by internal funds of the Fondazione IRCCS Ca' Granda Ospedale Maggiore Policlinico and by Ministry of Health Young Researcher grant no. GR-2011-0235287.

### Conflict of interest statement

The authors declare that the research was conducted in the absence of any commercial or financial relationships that could be construed as a potential conflict of interest. SM, SM-S, MV, RF, FM, SB, and AP are founders and shareholders of Newronika srl, Milan, Italy, a spin-off company of the Fondazione IRCCS Ca'; Granda Ospedale Maggiore Policlinico and of the University of Milan, Milan, Italy.

## References

[B1] Alusik S. Jedlickova V. Paluch Z. Zecova S. (2008). Plasma levels of nitrite/nitrate and inflammation markers in elderly individuals. Bratisl. Lek. Listy 109, 289–292. 18792481

[B2] Amatachaya A. Jensen M. P. Patjanasoontorn N. Auvichayapat N. Suphakunpinyo C. Janjarasjitt S.. (2015). The short-term effects of transcranial direct current stimulation on electroencephalography in children with autism: a randomized crossover controlled trial. Behav. Neurol. 2015, 1–11. 10.1155/2015/928631 25861158 PMC4377433

[B3] Anghinah R. Kanda P. A. Jorge M. S. Lima E. E. Pascuzzi L. Melo A. C. (2000). [Alpha band coherence analysis of EEG in healthy adult's and Alzheimer's type dementia patients]. Arq. Neuropsiquiatr. 58, 272–275. 10.1590/S0004-282X2000000200011 10849626

[B4] Biegon A. Eberling J. L. Richardson B. C. Roos M. S. Wong S. T. Reed B. R.. (1994). Human corpus callosum in aging and Alzheimer's disease: a magnetic resonance imaging study. Neurobiol. Aging 15, 393–397. 10.1016/0197-4580(94)90070-1 7969715

[B5] Bocci T. Marceglia S. Vergari M. Cognetto V. Cogiamanian F. Sartucci F.. (2015). Transcutaneous spinal direct current stimulation modulates human corticospinal system excitability. J. Neurophysiol. 114, 440–446. 10.1152/jn.00490.2014 25925328 PMC4509392

[B6] Boggio P. S. Ferrucci R. Mameli F. Martins D. Martins O. Vergari M.. (2012). Prolonged visual memory enhancement after direct current stimulation in Alzheimer's disease. Brain Stimulat. 5, 223–230. 10.1016/j.brs.2011.06.006 21840288

[B7] Boggio P. S. Khoury L. P. Martins D. C. S. Martins O. E. M. S. de Macedo E. C. Fregni F. (2009). Temporal cortex direct current stimulation enhances performance on a visual recognition memory task in Alzheimer disease. J. Neurol. Neurosurg. Psychiatry 80, 444–447. 10.1136/jnnp.2007.141853 18977813

[B8] Braak H. Braak E. Yilmazer D. de Vos R. A. Jansen E. N. Bohl J. (1996). New aspects of pathology in Parkinson's disease with concomitant incipient Alzheimer's disease. J. Neural Transm. Suppl. 48, 1–6. 10.1007/978-3-7091-7494-4_1 8988457

[B9] Chiaramonti R. Muscas G. C. Paganini M. Müller T. J. Fallgatter A. J. Versari A.. (1997). Correlations of topographical EEG features with clinical severity in mild and moderate dementia of Alzheimer type. Neuropsychobiology 36, 153–158. 10.1159/000119375 9313246

[B10] Cogiamanian F. Vergari M. Pulecchi F. Marceglia S. Priori A. (2008). Effect of spinal transcutaneous direct current stimulation on somatosensory evoked potentials in humans. Clin. Neurophysiol. 119, 2636–2640. 10.1016/j.clinph.2008.07.249 18786856

[B11] De Santi S. de Leon M. J. Rusinek H. Convit A. Tarshish C. Y. Roche A.. (2001). Hippocampal formation glucose metabolism and volume losses in MCI and AD. Neurobiol. Aging 22, 529–539. 10.1016/S0197-4580(01)00230-5 11445252

[B12] Delorme A. Makeig S. (2004). EEGLAB: an open source toolbox for analysis of single-trial EEG dynamics including independent component analysis. J. Neurosci. Methods 134, 9–21. 10.1016/j.jneumeth.2003.10.009 15102499

[B13] Duffy F. H. Albert M. S. McAnulty G. (1984). Brain electrical activity in patients with presenile and senile dementia of the Alzheimer type. Ann. Neurol. 16, 439–448. 10.1002/ana.410160404 6497353

[B14] Ewers M. Frisoni G. B. Teipel S. J. Grinberg L. T. Amaro E. Jr. Heinsen H.. (2011). Staging Alzheimer's disease progression with multimodality neuroimaging. Prog. Neurobiol. 95, 535–546. 10.1016/j.pneurobio.2011.06.004 21718750 PMC3223355

[B15] Farkas E. Luiten P. G. (2001). Cerebral microvascular pathology in aging and Alzheimer's disease. Prog. Neurobiol. 64, 575–611. 10.1016/S0301-0082(00)00068-X 11311463

[B16] Feng Y. Wang X. (2012). Antioxidant therapies for Alzheimer's disease. Oxid. Med. Cell. Longev. 2012:472932. 10.1155/2012/472932. 22888398 PMC3410354

[B17] Ferrucci R. Mameli F. Guidi I. Mrakic-Sposta S. Vergari M. Marceglia S.. (2008). Transcranial direct current stimulation improves recognition memory in Alzheimer disease. Neurology 71, 493–498. 10.1212/01.wnl.0000317060.43722.a3 18525028

[B18] Fonseca L. C. Tedrus G. M. A. S. Rezende A. L. R. A. Giordano H. F. (2015). Coherence of brain electrical activity: a quality of life indicator in Alzheimer's disease? Coerência da atividade elétrica cerebral: indicador da qualidade de vida na doença de Alzheimer? Arq. Neuropsiquiatr. 73, 396–401. 10.1590/0004-282X20150035 26017204

[B19] Gudmundsson S. Runarsson T. P. Sigurdsson S. Eiriksdottir G. Johnsen K. (2007). Reliability of quantitative EEG features. Clin. Neurophysiol. 118, 2162–2171. 10.1016/j.clinph.2007.06.018 17765604

[B20] Guix F. X. Uribesalgo I. Coma M. Muñoz F. J. (2005). The physiology and pathophysiology of nitric oxide in the brain. Prog. Neurobiol. 76, 126–152. 10.1016/j.pneurobio.2005.06.001 16115721

[B21] Guze B. H. Hoffman J. M. Baxter L. R. Mazziotta J. C. Phelps M. E. (1991). Functional brain imaging and Alzheimer-type dementia. Alzheimer Dis. Assoc. Disord. 5, 215–230. 10.1097/00002093-199100540-00001 1781964

[B22] Haxby J. V. Grady C. L. Friedland R. P. Rapoport S. I. (1987). Neocortical metabolic abnormalities precede nonmemory cognitive impairments in early dementia of the Alzheimer type: longitudinal confirmation. J. Neural Transm. Suppl. 24, 49–53. 3479529

[B23] Hogan M. J. Swanwick G. R. J. Kaiser J. Rowan M. Lawlor B. (2003). Memory-related EEG power and coherence reductions in mild Alzheimer's disease. Int. J. Psychophysiol. 49, 147–163. 10.1016/S0167-8760(03)00118-1 12919717

[B24] Jelic V. Johansson S. E. Almkvist O. Shigeta M. Julin P. Nordberg A.. (2000). Quantitative electroencephalography in mild cognitive impairment: longitudinal changes and possible prediction of Alzheimer's disease. Neurobiol. Aging 21, 533–540. 10.1016/S0197-4580(00)00153-6 10924766

[B25] Jeong J. (2004). EEG dynamics in patients with Alzheimer's disease. Clin. Neurophysiol. 115, 1490–1505. 10.1016/j.clinph.2004.01.001 15203050

[B26] Jiang Z. (2005). Abnormal cortical functional connections in Alzheimer's disease: analysis of inter- and intra-hemispheric EEG coherence. J. Zhejiang Univ. Sci. B 6, 259–264. 10.1631/jzus.2005.B0259 15754423 PMC1389734

[B27] Karas G. B. Burton E. J. Rombouts S. A. R. B. van Schijndel R. A. O'Brien J. T. Scheltens P. H.. (2003). A comprehensive study of gray matter loss in patients with Alzheimer's disease using optimized voxel-based morphometry. Neuroimage 18, 895–907. 10.1016/S1053-8119(03)00041-7 12725765

[B28] Katusic Z. S. Austin S. A. (2014). Endothelial nitric oxide: protector of a healthy mind. Eur. Heart J. 35, 888–894. 10.1093/eurheartj/eht544 24357508 PMC3977136

[B29] Kesslak J. P. Nalcioglu O. Cotman C. W. (1991). Quantification of magnetic resonance scans for hippocampal and parahippocampal atrophy in Alzheimer's disease. Neurology 41, 51–54. 10.1212/WNL.41.1.51 1985296

[B30] Klimesch W. (1999). EEG alpha and theta oscillations reflect cognitive and memory performance: a review and analysis. Brain Res. Brain Res. Rev. 29, 169–195. 10.1016/S0165-0173(98)00056-3 10209231

[B31] Koberda J. L. Moses A. Koberda P. Koberda L. (2013). Clinical advantages of quantitative electroencephalogram (QEEG)-electrical neuroimaging application in general neurology practice. Clin. EEG Neurosci. 44, 273–285. 10.1177/1550059412475291 23536380

[B32] Kramer M. A. Chang F. L. Cohen M. E. Hudson D. Szeri A. J. (2007). Synchronization measures of the scalp electroencephalogram can discriminate healthy from Alzheimer's subjects. Int. J. Neural Syst. 17, 61–69. 10.1142/S0129065707000932 17565502

[B33] Lamy J. C. Boakye M. (2013). BDNF Val66Met polymorphism alters spinal DC stimulation-induced plasticity in humans. J. Neurophysiol. 110, 109–116. 10.1152/jn.00116.2013 23576701

[B34] Leuchter A. F. Newton T. F. Cook I. A. Walter D. O. Rosenberg-Thompson S. Lachenbruch P. A. (1992). Changes in brain functional connectivity in Alzheimer-type and multi-infarct dementia. Brain J. Neurol. 115(Pt 5), 1543–1561. 10.1093/brain/115.5.1543 1422803

[B35] Liebetanz D. Koch R. Mayenfels S. König F. Paulus W. Nitsche M. A. (2009). Safety limits of cathodal transcranial direct current stimulation in rats. Clin. Neurophysiol. 120, 1161–1167. 10.1016/j.clinph.2009.01.022 19403329

[B36] Locatelli T. Cursi M. Liberati D. Franceschi M. Comi G. (1998). EEG coherence in Alzheimer's disease. Electroencephalogr. Clin. Neurophysiol. 106, 229–237. 10.1016/S0013-4694(97)00129-6 9743281

[B37] McKhann G. Drachman D. Folstein M. Katzman R. Price D. Stadlan E. (1984). Clinical diagnosis of Alzheimer's disease: report of the NINCDS-ADRDA work group under the auspices of Department of Health and Human Services Task Force on Alzheimer's Disease. Neurology 34, 939–944. 10.1212/WNL.34.7.939 6610841

[B38] Migliaccio R. Agosta F. Possin K. L. Rabinovici G. D. Miller B. L. Gorno-Tempini M. L. (2012). White matter atrophy in Alzheimer's disease variants. Alzheimers Dement. 8 5 Suppl., S78–S87.e1-2. 10.1016/j.jalz.2012.04.010 23021625 PMC3717610

[B39] Neuling T. Wagner S. Wolters C. H. Zaehle T. Herrmann C. S. (2012). Finite element model predicts current density distribution for clinical applications of tDCS and tACS. Front. Psychiatry 3:83. 10.3389/fpsyt.2012.00083 23015792 PMC3449241

[B40] Nitsche M. A. Paulus W. (2000). Excitability changes induced in the human motor cortex by weak transcranial direct current stimulation. J. Physiol. 527(Pt 3), 633–639. 10.1111/j.1469-7793.2000.t01-1-00633.x 10990547 PMC2270099

[B41] Nitsche M. A. Paulus W. (2001). Sustained excitability elevations induced by transcranial DC motor cortex stimulation in humans. Neurology 57, 1899–1901. 10.1212/WNL.57.10.1899 11723286

[B42] Paulus W. (2004). Outlasting excitability shifts induced by direct current stimulation of the human brain. Suppl. Clin. Neurophysiol. 57, 708–714. 10.1016/S1567-424X(09)70411-8 16106673

[B43] Priori A. (2003). Brain polarization in humans: a reappraisal of an old tool for prolonged non-invasive modulation of brain excitability. Clin. Neurophysiol. 114, 589–595. 10.1016/S1388-2457(02)00437-6 12686266

[B44] Pulgar V. M. (2015). Direct electric stimulation to increase cerebrovascular function. Front. Syst. Neurosci. 9:54. 10.3389/fnsys.2015.00054 25870543 PMC4378276

[B45] Scarpini E. Cogiamanian F. (2003). Alzheimer's disease: from molecular pathogenesis to innovative therapies. Expert Rev. Neurother. 3, 619–630. 10.1586/14737175.3.5.619 19810962

[B46] Selley M. L. (2003). Increased concentrations of homocysteine and asymmetric dimethylarginine and decreased concentrations of nitric oxide in the plasma of patients with Alzheimer's disease. Neurobiol. Aging 24, 903–907. 10.1016/S0197-4580(03)00007-1 12928048

[B47] Stevens A. Kircher T. Nickola M. Bartels M. Rosellen N. Wormstall H. (2001). Dynamic regulation of EEG power and coherence is lost early and globally in probable DAT. Eur. Arch. Psychiatry Clin. Neurosci. 251, 199–204. 10.1007/s004060170027 11829205

[B48] Trivedi D. P. Hallock K. J. Bergethon P. R. (2013). Electric fields caused by blood flow modulate vascular endothelial electrophysiology and nitric oxide production. Bioelectromagnetics 34, 22–30. 10.1002/bem.21741 22674251 PMC3522793

[B49] Truini A. Vergari M. Biasiotta A. La Cesa S. Gabriele M. Di Stefano G.. (2011). Transcutaneous spinal direct current stimulation inhibits nociceptive spinal pathway conduction and increases pain tolerance in humans. Eur. J. Pain 15, 1023–1027. 10.1016/j.ejpain.2011.04.009 21576030

[B50] Ulam F. Shelton C. Richards L. Davis L. Hunter B. Fregni F. (2015). Cumulative effects of transcranial direct current stimulation on EEG oscillations and attention/working memory during subacute neurorehabilitation of traumatic brain injury. Clin. Neurophysiol. 126, 486–496. 10.1016/j.clinph.2014.05.015 24947595

[B51] Vecchio F. Babiloni C. Lizio R. Fallani F. D. V. Blinowska K. Verrienti G.. (2013). Resting state cortical EEG rhythms in Alzheimer's disease: toward EEG markers for clinical applications: a review. Suppl. Clin. Neurophysiol. 62, 223–236. 10.1016/B978-0-7020-5307-8.00015-6 24053043

[B52] Vecchio F. Miraglia F. Quaranta D. Granata G. Romanello R. Marra C.. (2016). Cortical connectivity and memory performance in cognitive decline: A study via graph theory from EEG data. Neuroscience 316, 143–150. 10.1016/j.neuroscience.2015.12.036 26724581

[B53] Wang R. Wang J. Yu H. Wei X. Yang C. Deng B. (2014). Decreased coherence and functional connectivity of electroencephalograph in Alzheimer's disease. Chaos 24, 033136. 10.1063/1.4896095 25273216

[B54] Wassermann E. M. Grafman J. (2005). Recharging cognition with DC brain polarization. Trends Cogn. Sci. 9, 503–505. 10.1016/j.tics.2005.09.001 16182596

[B55] Welch P. (1967). The use of fast Fourier transform for the estimation of power spectra: A method based on time averaging over short, modified periodograms. IEEE Trans. Audio Electroacoustics 15, 70–73. 10.1109/TAU.1967.1161901

[B56] Zhu X. Smith M. A. Honda K. Aliev G. Moreira P. I. Nunomura A.. (2007). Vascular oxidative stress in Alzheimer disease. J. Neurol. Sci. 257, 240–246. 10.1016/j.jns.2007.01.039 17337008 PMC1952687

